# Effect of Cold on Giant Powder

**Published:** 1881-09

**Authors:** 


### EFFECT OF COLD ON GIANT POWDER.

Giant powder freezes at about 44 deg. Fahr., and gradually becomes hard, when it cannot be exploded with the giant powder caps. When received at a mine and is not already frozen, it should be kept in some warm place, where it cannot freeze, to avoid the trouble of thawing it. When the powder is frozen it should never be used for blasting. The powder may be in a granulated state, apparently soft to the touch, and yet the nitro-glycerine (the explosive property of the powder) be so chilled that its strength is only partially developed by an explosion. Frozen powder, when confined in a drill-hole, can be exploded by using a heavy primer of thoroughly thawed giant powder No. 1. Knowing this fact, it is foolhardy for men to try to pick out a charge of frozen powder from a drill hole, as it is always more or less attended with danger.

The Giant Powder Co. issue the following instructions as to the best methods of thawing the powder when frozen:

1. Put a layer of cartridges of frozen powder in a box partially filled with hot ashes, or sand, of about 100 deg. Fahr., and cover them well with the same material. Many miners prefer this to any other method for thawing powder.

2. Put a layer of cartridges in a wooden box and place the box near the boiler in the hoisting works or mill, or other warm place, turning the cartridges, from time, until the powder becomes soft and warm. It is then ready for use. The warmer the powder is when used, the greater the execution of the blast. After being well thawed it should be used at once before getting cold again.

3. Miners, by placing several cartridges in their boot legs, when going to work, will find the natural warmth of their bodies sufficient to thaw the powder, while they are "striking in a hole."

4. During the winter months, keep the powder in a *warm*, dry room, on shelves or slats ; when once thawed out, it will remain so.

The latest improvement in this direction is simply a jacketed chamber containing shelves on which the cartridges are laid, a small furnace underneath heating the water in the jacket. By this means the cartridges can never be heated above the boiling point of water, and there is no danger of explosion. The Giant Powder Company are now making these little furnaces for use at mines and on railroads. One large railroad in Oregon is now using some fifty of these thawing furnaces. They are easily carried along, and a fire can be built in them right where they stand, wherever that may happen to be. At mines the furnace could be placed in any convenient position.

